# 

**Published:** 1889-06-15

**Authors:** 


### THE HOSPITAL

A WEEKLY INSTITUTIONAL JOURNAL OF
Science, Medicine, Nursing & Philanthropy.

SATURDAY, JUNE 15th, 1889.

**The Clergy** - 159
**The "Bone of Contention"** - 160
**A New Lease of Life**:—A Family Complaint; Weary of Waiting; The Rendall Bequest; An Audacious Proposal; Is Consumption Contagious? The Value of Air, 161—Is Consumption Curable? Take it in Time; Extending the Field; An Entertaining Room; The Worth of Amusement; How to Get In, 162—Is the Money Wanted? Try All Things; A Reason for Preference; Seeing for Oneself; Free-will or Force; How the Wrong People Get In, 163—The Compressed Air Chamber; What It Does; In the Dispensary; Refreshments; Obstinate Patients, 164—Patients from all Quarters; Pianos and Pictures; Treasures in Canvas; The Patients' Verdict; The Banjo, 165—In the Gallery; Woman's Privilege; In the Wards; Memorial Beds, 166—A Touch of Chivalry; A Patient of Another Class; A Sad Story; The Separate System, 167—Give Him a Chance; In the Kitchen; Gas or Coal; Clouds and their Linings; Helen West, 168—A Samaritan Fund; The Question of Creed; An Object Lesson; No Room; The Demand and the Supply; Results - 169
**Special Notices for Hospital Sunday, 1889** -
**A Year's Work in the Hospitals and Medical Charities of London** - 171

EXTRA SUPPLEMENT—THE NURSING MIRROR.

**En Passant.**—Nurses as Guests—A Jubilee Chaplain—Princess Louise—A Nurse Collector—A Vigorous Matron—Registration of Midwives—Bangor Institution—Summer Afternoons—A Census of Hospital Workers during 1888 - xxxix
**Nurses and Registration** - xl
**Notes from Australia** - xl
**Some New Wound Dressings** - xli
**Everybody's Opinion.**—Nurses at Newcastle - xlii
**Vacancies.—Appointments** - xlii

